# Cell-Free Tumor DNA (cf-tDNA) Liquid Biopsy: Current Methods and Use in Brain Tumor Immunotherapy

**DOI:** 10.3389/fimmu.2022.882452

**Published:** 2022-04-06

**Authors:** Jack Wadden, Karthik Ravi, Vishal John, Clarissa May Babila, Carl Koschmann

**Affiliations:** Department of Pediatric Hematology and Oncology, Michigan Medicine, Ann Arbor, MI, United States

**Keywords:** liquid biopsy, glioma, immunotherapy, cell-free tumor DNA (cf-tDNA), Csf, plasma

## Abstract

Gliomas are tumors derived from mutations in glial brain cells. Gliomas cause significant morbidity and mortality and development of precision diagnostics and novel targeted immunotherapies are critically important. Radiographic imaging is the most common technique to diagnose and track response to treatment, but is an imperfect tool. Imaging does not provide molecular information, which is becoming critically important for identifying targeted immunotherapies and monitoring tumor evolution. Furthermore, immunotherapy induced inflammation can masquerade as tumor progression in images (pseudoprogression) and confound clinical decision making. More recently, circulating cell free tumor DNA (cf-tDNA) has been investigated as a promising biomarker for minimally invasive glioma diagnosis and disease monitoring. cf-tDNA is shed by gliomas into surrounding biofluids (e.g. cerebrospinal fluid and plasma) and, if precisely quantified, might provide a quantitative measure of tumor burden to help resolve pseudoprogression. cf-tDNA can also identify tumor genetic mutations to help guide targeted therapies. However, due to low concentrations of cf-tDNA, recovery and analysis remains challenging. Plasma cf-tDNA typically represents <1% of total cf-DNA due to the blood-brain barrier, limiting their usefulness in practice and motivating the development and use of highly sensitive and specific detection methods. This mini review summarizes the current and future trends of various approaches for cf-tDNA detection and analysis, including new methods that promise more rapid, lower-cost, and accessible diagnostics. We also review the most recent clinical case studies for longitudinal disease monitoring and highlight focus areas, such as novel accurate detection methodologies, as critical research priorities to enable translation to clinic.

## Introduction

Gliomas are a diverse set of brain tumors derived from glial brain cells. While a relatively small fraction of total cancer deaths per year, Gliomas cause significant morbidity and mortality with a five year survival rate as low as 7.2% depending on the tumor subtype (1). Current treatment options are mostly limited to surgical resection and chemoradiation, however, immunotherapy has recently been evaluated as an exciting new therapy to combat this disease (2–6).

Effective immunotherapy relies on an accurate diagnosis to guide treatment selection, and disease monitoring to identify if the glioma is responding to treatment or progressing. Because of their sensitive location in the brain, repeat biopsies are not feasible (7). Thus, radiographic imaging is commonly used for both initial diagnosis and disease monitoring. However, these images can be difficult to interpret due to various factors such as immunotherapy-induced swelling, leading to incorrect assumptions about a tumor’s response to treatment (8–12); a classic example is “pseudoprogression”, where immunotherapy-induced swelling is misinterpreted as tumor progression. Especially for immunotherapy response monitoring, which has a minimum four week iRECIST monitoring interval (13), misinterpretation can lead to unnecessarily long treatment and improper or delayed course correction (10). Additionally, imaging does not capture molecular information, which is becoming increasingly important for proper diagnosis (14, 15), identification of personalized targeted immunotherapies (16), and monitoring of tumor evolution to detect resistance mutations (17, 18).

To address these issues, liquid biopsies have emerged as a promising new diagnostic and disease monitoring approach for gliomas. Liquid biopsies work by recovering and quantifying tumor-related biomarkers shed by dying tumor cells into surrounding biofluids. Various studies have shown that biomarker levels correlate with tumor burden, and/or disease state, and may even be able to detect disease progression before it is evident in imaging (19, 20). Thus, liquid biopsies promise a minimally invasive and accurate alternative diagnostic to tissue biopsy, and a less error prone approach to quantify tumor response than radiographic imaging (13).

Circulating tumor cells from primary brain tumors have been identified in blood (21, 22), however since primary brain tumors rarely metastasize, these cells are exceptionally rare (23). In this mini-review, we focus on the use of cell-free tumor DNA (cf-tDNA) as a biomarker for gliomas and its potential to aid in development and clinical use of immunotherapies targeting gliomas. We first summarize the mechanism of glioma cf-tDNA release. We then discuss both established and novel cf-tDNA detection methods used in the literature and their strengths and weaknesses. We then discuss translational uses of cf-tDNA liquid biopsies in clinic focusing on efforts to improve immunotherapy-based treatment. Finally, we discuss current difficulties and open questions about the practical use of liquid-biopsy and new approaches to cf-tDNA detection that attempt to improve accuracy, accessibility, and cost.

## cf-tDNA Liquid Biopsy in Gliomas: an Overview and Key Principles

**Figure 1:**
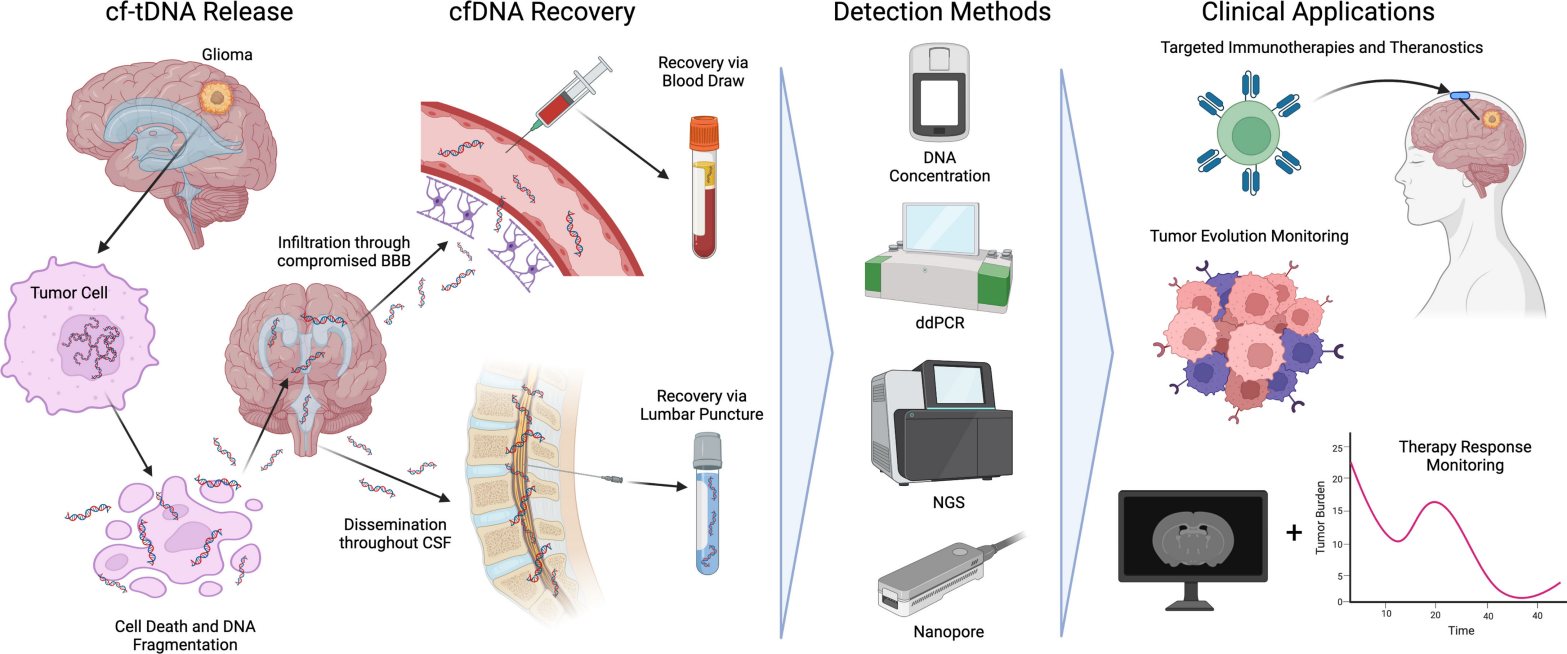
An overview of cf-tDNA release into biofluids, recovery, detection methods, and current clinical applications benefiting immunotherapies for glioma.

As glioma cells proliferate and die *via* apoptosis, necrosis, or immune response, tumor DNA is immediately shed into the surrounding interstitial fluid and CSF. During apoptosis, tumor chromosomal DNA is fragmented *via* endonucleases around nucleosome boundaries (~140bp-180bp) resulting in a characteristic pattern of fragmentation (24). cf-tDNA fragments spread throughout the central nervous system before eventually permeating the blood-brain barrier (25). Due to the low molecular weight of post-apoptotic cf-tDNA, the molecules are more able to permeate selective filters in the body such as the blood-brain barrier and glomerulus structures in the kidneys. To date, glioma cf-tDNA has been successfully identified in CSF, plasma, and even urine (24). **Figure 1** shows an overview of cf-tDNA release, recovery, current detection methods, and clinical applications.

cf-tDNA signals can be distinguished from background cfDNA (from non-tumor tissue) by using either aggregate or specific detection techniques. Aggregate detection relies on biomarkers that are shared by both healthy and tumor-derived DNA but are up- or down-regulated in tumors. Prior work has detected glioma *via* structural variation, copy number alterations (26), methylation status of certain genomic regions (25, 27), and even cfDNA fragmentation patterns (24). However, these aggregate signals cannot uniquely discriminate tumor- vs. normal-derived cfDNA and are thus less likely to be useful when the relative amount of cf-tDNA is low (e.g., <1%). A more precise approach is to directly identify cf-tDNA by detection of genetic tumor driver mutations *via* probe-based quantitative PCR or cfDNA sequencing. These assays report the ratio of mutated to total cfDNA reads – i.e., the mutant or variant allele fraction (MAF/VAF) –identified in the sample.

Recovered cf-tDNA concentrations can vary widely and have been found to correlate with variables such as disease grade (28), tumor burden, tumor location relative to CSF reservoirs (29) and biofluid proximity to the tumor (30). Due to the highly-selective nature of the blood brain barrier, glioma cf-tDNA concentrations are generally several orders of magnitude higher in CSF than plasma (31) or urine (24), where typical plasma VAFs are <1% (17, 32) with suspected positives detected as low as 0.02% (32). Thus, CSF is considered the gold standard biofluid for liquid biopsy of gliomas. However, lumbar punctures to obtain CSF are significantly more invasive than blood draws and urine collection, making plasma and urine-based liquid biopsies much more desirable. This motivates ultra-sensitive detection techniques to enable accurate monitoring of both CSF-derived cf-tDNA and allow for practical use of plasma and urine-based biopsies.

## Cf-tDNA Detection Methods

Multiple cf-tDNA detection methods have been used to successfully quantify cf-tDNA related biomarkers and uncover diagnostically relevant information that might guide personalized treatment. In this section we review popular cf-tDNA detection methods and their benefits and weaknesses.

### Cell-Free DNA Concentration and Other Methods

Total cfDNA concentration is the level/amount of DNA per volume of biofluid (blood, CSF, or urine). Because tumor cell turnover is higher than that of normal tissue, research has found that glioma patients tend to have higher absolute amounts of cfDNA in glioma than healthy patients (24, 33–35). However, total cfDNA can be impacted by many other factors unrelated to tumor burden (e.g. inflammation) reducing its sensitivity to detect disease without supplementary analysis (36, 37). Furthermore, concentration as a biomarker lacks molecular information that might inform targeted treatments and clinical management. Methylation of cf-DNA and recovery *via* methylation-specific PCR (27) or sequencing (38) has also been proposed as method of detecting disease *via* measurement of hypo/hyper-methylation at various genomic loci but is not discussed in this review.

### Droplet Digital PCR (ddPCR)

qPCR (quantitative polymerase chain reaction) is a quantification method that uses sequence specific primers or fluorescent probes to detect and quantify tumor-specific somatic mutations (39). However, qPCR suffers from a variety of shortcomings that limit its sensitivity and specificity (40–42). Droplet digital PCR (ddPCR) is a modification of qPCR that improves precision and limit-of-detection (42). By dividing a typical qPCR reaction into many isolated droplets with ~1 template copy, a precise VAF can be computed from the ratio of mutant positive to wildtype droplets, with a VAF limit of detection around 0.001% (43). ddPCR has supplanted itself as a highly-accurate technique, and is considered a gold standard approach to quantify VAFs from liquid samples (36, 44). However, accurate and reproducible ddPCR assays require careful development and optimization of input template concentration, target-specific primers, and fluorescent probes and are restricted for use on a limited set of known hotspot mutations (44).

### Next-Generation Sequencing

Next-generation sequencing (NGS) refers to a group of massively parallel sequencing technologies including Ion Torrent (45, 46), PacBio (47), and Illumina (48, 49), with the latter used most often for liquid biopsy due to its accuracy (50). Illumina sequencing uses synthesis of fluorescent dNTPs to clusters of template strands to recover the original template sequence (45). Unlike ddPCR, sequencing of cf-tDNA does not rely on sequence specific probes or any prior knowledge of mutations. Various library preparations enable sequencing of the whole genome (WGS), whole exome (WES), targeted hybridization capture, or amplicons from targeted panels with each technique trading genome coverage for read depth. For example, WGS can provide 20x-50x coverage over the entire genome, which enables detection of high-frequency somatic mutations and copy number variation but is too shallow to precisely measure cf-tDNA allele fractions below ~2-5%. Targeted amplicon sequencing can generate >10,000x coverage of specific genomic loci, improving VAF limit of detection but reducing the number of analyzed loci. Illumina sequencing has proven highly-accurate, with an error rate ranging between 0.5%-1% (50–52). However, this approach is relatively expensive, slow, and due to its error rate, cannot reliably detect allele fractions below the sequencer error rate without further assay modifications (53–56).

### Nanopore Sequencing

Nanopore sequencing is a relatively new technology (57) that has been used for liquid biopsies (58, 59). Nanopore sequencers work by feeding DNA strands through small pores embedded in a membrane. As they flow through the pore, each DNA base-pair creates a unique electrical disturbance that can be measured and used to call each base. Nanopore devices are low-cost, can sequence any length DNA strand, have a small form factor, and offer a rapid time to result making them ideal devices for liquid biopsy. However, the device’s error rate has traditionally prevented it from being applied to liquid samples where allele frequencies are less than ~2% (58). Our group previously analyzed CSF samples from 12 pediatric high grade glioma patients and found that nanopore had a 85% sensitivity and 100% specificity in CSF samples (58), which compared favorably to Illumina-based targeted sequencing. More recent improvements to basecaller accuracy, and also the use of circular consensus sequencing (59–61) have improved accuracy to <0.05%, comparable with ddPCR-based approaches (59).

While not perfect, these cf-tDNA detection methods have been used to demonstrate a variety of potential uses for glioma diagnosis and monitoring. In the next section, we highlight translational research that attempts to utilize these instruments to improve disease management.

## Clinical Applications of Liquid Biopsy for Immunotherapy

Accurate cf-tDNA-based liquid biopsies have several promising clinical applications for immunotherapy. Here, we highlight recent translational research (also summarized in **Table 1**) attempting to use liquid biopsy diagnostics that could help guide the use of personalized immunotherapy and monitor disease response in gliomas.

**Table 1:** A summary of cf-tDNA-based liquid biopsy detection methods as applied to gliomas and associated limit of detection, typical time-to-result, and cost.

	cfDNA Concentration		ddPCR		WES/WGS/low-depth capture NGS		High-depth, targeted NGS		Targeted Nanopore	
**Approximate VAF LoD**	–		0.001% (43)		~1-10%		>0.01%-0.02% (29, 32)		2%-5% (58)	
**Enhanced assay VAF LoD**	–		–		–		>1e-9 (54)		~0.001 (59)	
**Typical Time-to-result**	<1hr		~5hrs		3-21+ days		3-21+ days		1-2 days	
**Cost**	$		$		$$$-$$$$		$$$		$$-$$$	
**Diagnostic method**	up/down regulation		Fluorescent probe-based		Sequencing		Sequencing		Sequencing	
**Biofluid**	CSF	Blood	CSF	Blood	CSF	Blood	CSF	Blood	CSF	Blood
**Relevant Work in Glioma Diagnostics**	**-**	(36)	(62–67)	(18, 36, 62, 63, 67, 68)	(24, 26, 29, 64, 69–72)	(24, 70)	(29, 46, 58, 67)	(17, 32, 67, 73)	(58)	–
**Relevant Work in Glioma Monitoring**	**-**	–	(19, 30, 70, 74)	(19, 68, 70, 74)	(17, 72)	–	–	(35)	(58)	(59)*

ddPCR is an accurate, rapid, and cost-effective approach for both diagnostics and monitoring in both CSF and plasma, but it is limited by the number of mutations it can detect and track. NGS sequencing-based techniques can capture a wider variety of mutations, but their cost and typical time-to-result make them impractical for use in applications that require rapid turn-around times such as treatment response monitoring. Targeted Nanopore Sequencing coupled with enhanced assay design may offer the best path forward to accurate, affordable, and rapid disease characterization and monitoring. *Marcozzi et al. was not applied to gliomas but is considered relevant due to its potential utility (59). Time and cost metrics are highly variable and depend on the ability to batch samples, bulk purchasing price reductions, target panel size, and available institutional resources. These estimates are based on our experience. $ = <$100 USD; $$ = $100-$500 USD; $$$ = $500-$1000 USD; $$$$ = > $1,000 USD.

### Personalized Diagnostics and Treatment Selection

Identifying tumor-specific molecular information in cf-tDNA that provides an accurate diagnosis, prognosis, and predicts response of a particular treatment is a “holy grail” clinical application for liquid biopsies. There is some work linking molecular markers (e.g. SNVs or CNVs) to the predicted response to radiation or chemotherapy in gliomas [reviewed in Birko et al. (75)]. However, minimal work has explored cf-tDNA diagnostics to personalize immunotherapy treatment. Studies looking at other solid tumors have identified several cell free DNA biomarkers as predictors of immunotherapy response (76), most notably increased tumor mutational burden (TMB) (77–79) and reduced copy number variations (80, 81). Pepe et al. showed feasibility for assessment of TMB in cytological samples from patients with NSCLC using a NGS platform (82). Studies have aimed to identify specific hotspot mutations that predict response to immune checkpoint inhibition or other immunotherapies. Guibert et al. identified that mutations in *KRAS* or *TP53* without *PTEN* loss lead to increased PD-L1 expression and increased tumor mutational burden, increasing response to PD-1 immune checkpoint inhibition (77). This work was done in lung cancer, but the aforementioned mutations are also commonly present in gliomas, raising a potential opportunity to use glioma cf-tDNA to predict immunotherapy efficacy.

### Tumor Evolution Monitoring

Another important clinical application of liquid biopsy for gliomas is the monitoring of tumor evolution. It has been shown that tumors undergo considerable evolution over the course of treatment, resulting in genetic changes that might suggest a new diagnosis and an adjustment to disease management (69). Several studies have investigated tumor evolution in glioma, with estimates ranging from 33-73% of genetic mutations at recurrence matching with alterations at biopsy (83, 84). However, as previously discussed, serial biopsies are discouraged due to the increased chance of morbidity. Miller et al. used CSF-derived cf-tDNA to monitor tumor evolution in adult gliomas (17) and was able to identify cf-tDNA in 42 of 85 patients who underwent CSF collection and NGS sequencing. In patients with hypermutated tumors, the median percentage match between the CSF derived cf-tDNA mutations and the initial tissue alterations was only 19.6%, while non-hypermutated tumors had an 81.7% match. These results indicate that CSF-based liquid biopsies can capture tumor heterogeneity and used to monitor tumor evolution over time. While this work was not applied to immunotherapy-based treatment, tumor evolution monitoring could be used to identify increased TMB and PD-1 sensitivity (77), or acquired resistance markers (85).

### Treatment Response Monitoring and Resolution of Pseudoprogression

In addition to tumor evolution, several studies have shown the utility of serial cf-tDNA sampling for treatment response monitoring and resolution of pseudoprogression in gliomas (19, 37, 58, 62, 68, 69, 74, 86). One of the largest studies thus far from Panditharatna et al. collected serial CSF samples and concordant MRI from 22 patients (74). They found that cf-tDNA decreased in response to radiotherapy in 83% of patients, which was corroborated by a decrease in tumor size on MRI. The first prospective high grade glioma clinical trial with serial liquid biopsy was recently published by our group (19). We collected serial CSF and plasma samples from 24 patients and found that patients with decreased H3K27M CSF and plasma cf-tDNA VAF had prolonged progression free survival (19). A similar trend was identified by Jensen et al. while tracking response to immunotherapy over a variety of cancers (20).

We also compared serial ct-DNA levels with corresponding radiographic imaging. In individual cases, they were able to identify instances of suspected pseudoprogression, where radiographic progression was accompanied by a decrease in cf-DNA VAF. In another patient, a large increase in cf-tDNA VAF (>25%) preceded radiographic progression in many patients, suggesting that cf-tDNA VAF changes may act as an earlier warning sign of tumor progression versus radiographic imaging. For immunotherapy-based response, Jensen et al. used shallow WGS (0.3x) of cf-DNA to identify copy number alterations (CNAs) and report a metric of “genome instability” over a variety of cancers (20). This study demonstrated that dynamic changes in CNAs could track immunotherapy response and were able to resolve pseudoprogression, but specific use in gliomas has yet to be demonstrated. A common theme among these studies is relative changes in cf-tDNA signals—rather than absolute values—are better indicators of tumor response. Taken together, these results reaffirm the potential clinical utility of serial liquid biopsies for improved molecular profiling and effective therapeutic monitoring for gliomas.

## Discussion

While exciting progress is being made developing liquid biopsies that can support immunotherapy-based treatment of gliomas, further work is required to improve understanding tumor-specific biomarker release and how it corresponds to tumor burden, improve detection accuracy of various assays, and investigate novel liquid biopsy approaches that offer improved sensitivity and specificity.

### Current Issues in Understanding cf-tDNA Release and Dissemination

Our current understanding of glioma cf-tDNA release and dissemination to various biofluids is still limited. Research has highlighted variability depending on a tumor’s proximity to CSF reservoirs in the brain (17, 69). This raises concerns about the ability of liquid biopsies to accurately track tumor burden if disease spreads. Blood-brain barrier permeability can also vary highly case-to-case, further complicating efforts to correlate tumor burden with cf-tDNA levels in blood. It is also unclear how various treatments (e.g. radiation, chemotherapy, and immunotherapy) impact both normal cfDNA and cf-tDNA release over time, which could bias cf-tDNA levels and VAF if not properly accounted for (10, 12). Future work might incorporate variables such as tumor ventricle proximity, tumor biology, and treatment type to better understand these patterns.

### Are Detection Method Precision and Accuracy Holding Back Plasma-Based Approaches?

Even though there is a general consensus that the sensitivity and specificity of CSF-based assays are superior to plasma (18, 28, 70, 87, 88), plasma- and urine-based liquid biopsies are still highly desirable due to the ease of sample collection. It is likely that the large disparity between CSF- and plasma-derived results are partly due to limitations of current gold standard detection methods. As an example of the difficulty of implementing precise detection methods, Li et al. measured the performance of multiple ddPCR-based assays for the detection of H3.3K27M mutations in matched tissue, CSF, and plasma samples across three independent labs using two commercially available ddPCR machines (44). Results indicated that ddPCR was capable of precisely measuring small VAFs from plasma-derived cfDNA, but discovered high-variation among replicates, and statistically significant differences across assays and ddPCR instrument vendors. Significant protocol optimizations were required to improve the sensitivity, repeatability, and reliability of the assay (44). When considering NGS detection methods, even the most accurate NGS instruments have an established raw error rate of ~0.1%, which is most likely too high to precisely resolve plasma-derived cf-tDNA levels that fluctuate between 1% and 0.05%. These results highlight the need for highly optimized and standardized versions of current approaches, as well as improved detection methods to enable proper translation to clinic.

### Future Directions

The current limitations with ddPCR, NGS, and Nanopore-based liquid biopsy approaches are not easy to solve, but progress is being made *via* improved assay design and bioinformatic error correction. For example, the use of universal molecular identifiers (UMIs) during targeted amplification can help resolve sequencing errors targeted amplification, enabling detection of 1 mutant molecule in 10,000 (89), with other assay design techniques further improving detection sensitivity by several orders of magnitude (53–56). Nanopore, long-read sequencing offers the ability to sequence single-molecule tandem-repeats constructed from small cf-tDNA fragments using rolling circle amplification. Even though the native error rate for Nanopore sequencing is relatively high (58), its ability to sequence long DNA strands with multiple redundant copies of a single cf-tDNA template allows for accuracy beyond any available NGS or ddPCR approach (59–61). Some of these methods are so accurate, that they are limited by polymerase error rather than sequencer error rate (59). Furthermore, continual improvements to basecalling software, library preparation methods, and assay design are certain to further reduce false positive rates. Because of these factors we expect long-read, consensus sequencing approaches to become a gold standard liquid biopsy approach for plasma cf-tDNA in the future.

Recent work has explored the effectiveness of CAR-T cell therapy in diffuse midline gliomas, administered serially into CSF *via* Ommaya reservoirs (6, 90). Ommaya reservoirs are used for intra-cranial administration of immunotherapies as well as frequent, minimally invasive recovery of CSF without the need for lumbar punctures. Ommaya reservoirs would allow for more practical, and frequent use of CSF to monitor disease and apply liquid biopsy techniques more frequently. More frequent sequencing-based liquid biopsies might add undue cost to treatment. This motivates use of lower-cost techniques such as ddPCR as well as investigation of cost-effective sequencing approaches like single-use Oxford Nanopore flow-cells (91).

## Author Contributions

JW designed the original paper. JW, KR, VJ, and CB were responsible for initial manuscript generation and editing. JW was responsible for final manuscript preparation. CK provided funding and editing. All authors contributed to the article and approved the submitted version.

## Funding

JW is supported by the National Institutes of Health under award number T32HL749, the University of Michigan Chad Carr Pediatric Brain Tumor Center, Catching Up With Jack, and the Pediatric Brain Tumor Foundation. CK is supported by NIH/NINDS Grant R01-NS124607 and R01-NS119231 and Department of Defense Grant CA201129P1, the University of Michigan Chad Carr Pediatric Brain Tumor Center, the ChadTough Defeat DIPG Foundation, the DIPG Collaborative, Catching Up With Jack, The Pediatric Brain Tumor Foundation, The Yuvaan Tiwari Memorial Foundation, The Morgan Behen Golf Classic, and the Michael Miller Memorial Foundation.

## Conflict of Interest

The authors declare that the research was conducted in the absence of any commercial or financial relationships that could be construed as a potential conflict of interest.

## Publisher’s Note

All claims expressed in this article are solely those of the authors and do not necessarily represent those of their affiliated organizations, or those of the publisher, the editors and the reviewers. Any product that may be evaluated in this article, or claim that may be made by its manufacturer, is not guaranteed or endorsed by the publisher.
